# Impact of ‘infodemic in pandemic’ on food and nutrition related perceptions and practices of Indian internet users

**DOI:** 10.1371/journal.pone.0266705

**Published:** 2022-04-21

**Authors:** SubbaRao M. Gavaravarapu, Ananya Seal, Paromita Banerjee, Thirupathi Reddy, Naresh Pittla

**Affiliations:** 1 Nutrition Information, Communication & Health Education (NICHE) Division, ICMR-National Institute of Nutrition, Hyderabad, Telangana, India; 2 Biostatistics Division, ICMR-National Institute of Nutrition, Hyderabad, Telangana, India; Universitá degli Studi di Milano, ITALY

## Abstract

The uncontrolled spread of (mis)information, news and propaganda related to COVID 19 created an ‘infodemic’ leading to panic and unscientific practices among the mass. With the largest number of internet users in the world, India has witnessed a steep rise in the number of people seeking information on social media related to COVID-19, which reached a staggering 22.3 million by March, 2020. This study aimed to evaluate the trend of COVID-19 associated food and nutrition news search by Indian internet users between 27^th^ January 2020 to 30^th^ June 2021 (time period between the first detected COVID-19 case and the end of the second wave in India) and its impact on their perceptions and practices. The association between the change in Relative Search Volume (RSV) on Google Trends (GT) of 34 popularly searched keywords classified by the researchers under 5 different categories—“Immunity”, “Eating behavior”, “Food safety”, “Food scares and concerns” and “Covid scare” showed a steep rise in search for immunity boosters, vitamin supplement brands “ayush kadha (ayurvedic decoction) during the first wave (April- August 2020). With a brief period of decline in the search trend, it again hiked correspondingly with the growing number of positive cases during the second wave in India. An online survey conducted on adult Indian internet users (n = 572) reported high (71.9%) consumption of Vitamin C rich fruits as well as Vitamin C (68.2%) and Zinc (61.4%) supplements to boost immunity. Traditional Indian spices like ginger and garlic were used by 62.9% and 50.9% respondents respectively. Most respondents reported to rely on social media for gathering COVID-19 associated tips for boosting immunity, however those with history of COVID-19 infection reported to rely more on doctors and health professionals for information. This study highlights the need of media and health literacy to advocate for the use of health information cautiously.

## Introduction

Even before the declaration of COVID-19 as a global pandemic by the World Health Organization (WHO) on March 11, 2020, the WHO Director-General, Tedros Adhanom Ghebreyesus had mentioned another epidemic, an epidemic of misinformation. It was termed as ‘Infodemic’, which literally means superfluity of information [1]. Though availability of information is essential for mankind, just as overabundance of junk food can lead to obesity overabundance of information, diet can lead to cluelessness [2]. The abundance of COVID-19 related information disseminated through an interconnection of sources such as the web, digital and social media platforms, radio, television, newspaper and other communication channels bewildered people [3]. Internet, the largest and the fastest platform for billions of people to seek health information [4] became a vital source of COVID-19 related information during the phases of imposed physical distancing and lockdowns, to control the spread of the virus. Internet usage had accelerated globally and data from Internet Service Providers in Europe reported 15–20% increase of internet traffic during the first few weeks of the pandemic [5]. In India too there has been an increase in the internet usage which is reflected by the amplified conversations about COVID-19 on social media reaching 22.3 million by the end of March, 2020 [1]. COVID-19 became the most trending and the most searched topic on Google as per Google Trends (GT) [6]. However, the quality of information available on the internet is unregulated and is likely to possess low-credibility. Thus, a lot of misinformation about the spread of COVID-19 as well as safety measures had made their way through the internet and social media creating confusion, panic and concern among internet users.

Although it is not a proven food borne pathogen, COVID-19 was associated with food from different prospects since its advent. Firstly, as the initial cases were being reported from a seafood market in Wuhan city, China [7, 8] there had been concerns about COVID-19 being transmitted through nonvegetarian foods or Chinese cuisine, food delivery, food packaging. In spite of limited evidence associated with survival of the virus on food surfaces or the risk of its transmission through ingestion of contaminated food [8, 9], in India the situation was no different. Information regarding ‘immunity-boosting’ food items as a preventive or therapeutic strategy against COVID-19 grabbed societal attention [9, 10]. This overabundance of information led to confusion and high interest in home remedies and self-medications [1]. Studies have reported increased consumption of certain micronutrients such as zinc, vitamin C, and vitamin A through increased intake of certain food items, nutraceuticals, and herbal traditional medicines [11, 12]. Also there have been reports on over-consumption of carbohydrate and fat dense food owing to quarantine related situational stress and boredom, increasing the risk of developing a dangerous vicious cycle of obesity and COVID-19 [13]. Overall, the COVID-19 pandemic appears to have impacted the general population’s food related behavior. Therefore, a study is designed to examine the web search behaviour of Indian internet users in relation to the top trending COVID- 19 associated food and nutrition news during the different phases of the pandemic. Also, to assess the impact of COVID-19 Infodemic on food and nutrition related perceptions and practices among Indian internet users and the extent of reliance on various sources of information.

## Materials and methods

The study was conducted in two distinct phases.

**I.** In the first phase, the most common food associated COVID-19 articles appearing on print and *e*-media between March-April 2020 were identified through manual search by two independent researchers. The commonly appearing phrases in the news were then categorized into 5 broad categories: “Immunity”, “Eating behavior”, “Food safety”, “Food scares and concerns” and “Covid scare” after discussion within the research team **(Table 1).** Each of these words/phrases was used as a keyword on Google Trends (GT) to obtain their Relative Search Volumes (RSV) for the period between 27^th^ January 2020 and 30^th^ June 2021, which encompassed the time period between the first detected COVID-19 case and the end of the second wave of infection in India. Since Google is the most popular mobile search engine in India with 99.68% share of the Indian market [14] its traffic data was taken to assess the search behavior of the users during the study period. The popularity of each search term and relevant changes over the period was associated with the reported number of COVID-19 cases in India during the corresponding period to understand the impact of COVID-19 scare on the behavior of the users. The COVID-19 case related data were obtained from the country specific WHO database [15].

**Table 1:** Categorization of search terms used as key words to get relative search volumes for the period (27^th^ Jan 2020 to 30^th^ June 2021).

IMMUNITY	EATING BEHAVIOUR	FOOD SAFETY	FOOD SCARE AND CONCREN	COVID SCARE
**NEUTRACEUTICAL/ SUPPLEMENTS** “vitamin d” “vitamin c” “limcee” “zincovit” “vitamin” “supplement” **ALTERNATIVE/ HOME REMEDIES** “AYUSH” “home remedy” “ayurvedic medicine” “gargle” “ayush kadha” “immunity booster drink” **NATURAL FOOD SOURCES** “green tea” “ginger” “turmeric” “fruits and vegetables”	“restaurant” “take away” “food delivery” “zomato” “swiggy” “grocery delivery” “street food” “junk food” “alcohol” “fried foods” “fruits and vegetables” “healthy food” “healthy recipes”	“vegetable cleaners” “wash vegetable”	“coronavirus in chicken” “healthy food” “healthy recipes”	“corona virus cases today” “covid cases” “confirmed cases in INDIA (WHO, report)”

**II.** In the second phase, a closed-ended questionnaire was developed, pre-tested and administered online to obtain cross-sectional information from active internet users in India regarding their perceptions, practices and the reliability of the commonly propagated food related information with respect to COVID-19. The participation of potential respondents was solicited through calls for participation issued via media releases, social media posts on the institute’s website and instant messaging apps. The survey form was made available on the official website and the links were shared on social media pages of Indian Council of Medical Research- National Institute of Nutrition (ICMR-NIN) between 1^st^ June 2021 to 31^st^ July 2021. Reminders for participation were sent out at regular intervals on all the above-mentioned media. Participants were eligible to take the survey if they were active internet users above the age of 18 who regularly accessed e-news and were residing in India. The study protocol was approved by the Institutional Ethical Committee of the ICMRNIN, Hyderabad, India. The e-participants had to provide electronic informed consent before taking the survey. Participants were asked to determine the changes incurred in their food safety practices, eating pattern during the COVID-19 period and about their knowledge, perception and perceived reliability on different sources of information: newspaper, television, social media, frontline health workers, health organizations, internet search or peer group.

### Questionnaire development

The survey questionnaire was designed using Yii2 Framework (PHP) and MySQL database was used to store the collected data. HTML, CSS, was used to design the front-end of the application. In order to make the questionnaire user friendly i.e., accessible on any device, screen size, platform and orientation Responsive Web Design (RWD) method was followed. Questions were all interlinked and dependent on respondent’s input, which was validated using JavaScript for generating the corresponding questions. The validation was done on the basis of the respondent’s age, internet usage, residence (as the survey questionnaire was made accessible exclusively for Indian IP addresses) to avoid inaccurate data. To avoid data redundancy, the survey application was designed to deny multiple submissions from the same page. The survey questionnaire consisted of two sections -first section included the brief outline about the survey, title of the survey, purpose and objectives of the survey. Second section contained 8 validation questions based on the inclusion criteria of the study. If the respondent was eligible for the survey, he/she was redirected directly to the main questionnaire. To protect the data privacy and reduce intrusion attacks the survey tool was hosted with Secure Sockets Layer (SSL) Certificate, a standard security technology for establishing an encrypted link between a server and a client.

The questionnaire consisted of 28 questions in 5 sections—Questions related to demography, internet usage, food safety measures taken during different phases of COVID-19, changes in perception and consumption of food during different phases of COVID and reliable sources of information were asked in the different categories. The questions dealt with time spent on internet to look for COVID-19 related information, procedures to wash vegetables, infection condition, use of special cleaners, use of online grocery and food delivery, changes in food choices and concerns developed against, sources of information on COVID-19 and perceived reliability were among the few information asked in the questionnaire.

Content validation of the questionnaire was done by 5 experts from different fields. Then the online questionnaire administration was piloted on 48 random participants using snowballing technique. Following this, some minor modifications were carried to the questionnaire before online administration.

### Statistical analysis

Descriptive analysis of change in the RSV of each of the 34 keywords during the study time frame (27^th^ January 2020 to 30^th^ June 2021) has been represented as time trends data. Karl- Pearson correlation coefficient was used to measure the strength of correlation between the RSVs of each keyword with weekly confirmed cases. The data from the country wide survey was analyzed using descriptive statistics to estimate the perception, practice and reliability of the study participants on the commonly propagated food related information with respect to COVID-19. The statistical analysis was done using R studio Connect 2021.09, IBM SPSS Statistics 24.

## Results

### I. Web search behaviour of Indian internet users in relation to the top trending COVID- 19 associated food and nutrition news: Common media propagated news and RSV of searches

**Fig 1:**
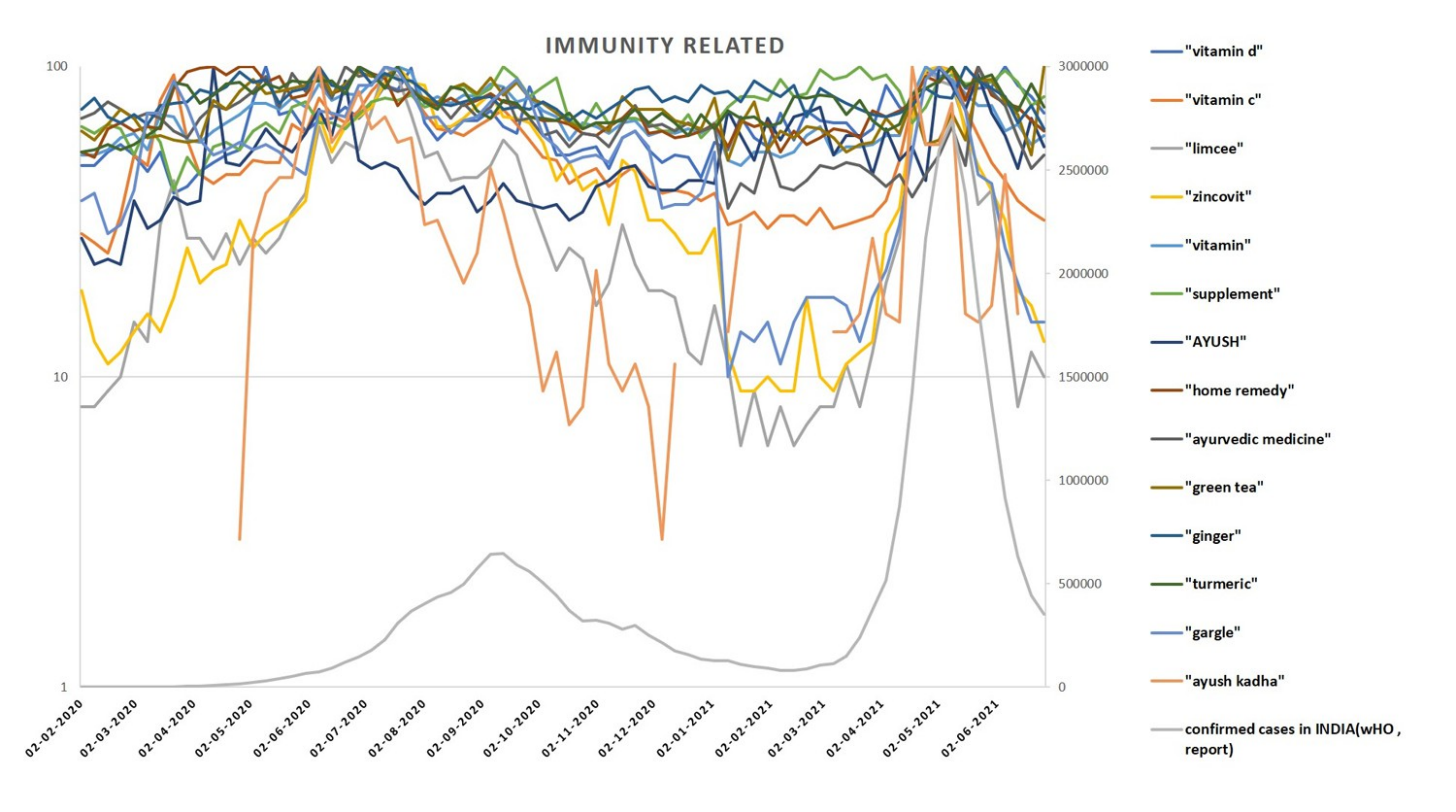
A positive correlation was observed between the number of COVID cases and search terms like “vitamin D”, “Limcee”, “grocery delivery”, “vegetable sanitizers”, “alcohol” etc. whereas, search terms like “street food”, “junk food”, “food delivery” etc. were negatively correlated to the number of cases.

**Fig 2:**
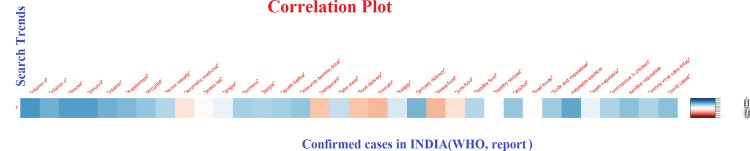
The correlation between the number of COVID cases and search terms (the darker the shade of blue the stronger is the correlation while the darker the shade of red the stronger negative correlation of each key word with the number of COVID cases).

The initial search about food associated COVID-19 news resulted in the following commonly appearing queries on Google: “Can I take vitamin supplements to prevent coronavirus?”, “vitamins to prevent corona”, “can I get coronavirus from eating frozen foods and ice cream?”,” herbal medicine to treat corona”. The categorization of the 34 selected keywords is given in **Table 1.** The Google Trends (GT) data of the selected keywords showed that the RSV of certain terms like “limcee” (over the counter Vitamin C supplement in India), “zincovit” (OTC zinc supplement), “ayush kadha”; “grocery delivery”; “wash vegetable”, “coronavirus in chicken” was high during April- August 2020. Whereas, terms like “food delivery”,” junk food”, were searched less during this period. The search trends declined with the decline in the number of positive cases in India but again hiked up during April- May 2021, as India was then hit by the second wave of COVID-19. The following figure shows the trend of search during the different phases of COVID-19 in India **Fig 1**. The correlation between the number of COVID cases and search terms is given in **Fig 2**.

### II. Impact of COVID-19 infodemic on the food and nutrition related perception, practices and reliability on the source of information among Indian internet users

The online questionnaire was attempted by 957 participants and after eliminating the ineligible participants (below 18 years or non-regular internet users or those who do not access e-news), duplicates and incomplete submissions, 572 were considered eligible for analysis. The demographic profile of the final respondents is given in **Table 2.** The subjects were from urban and rural areas covering all Indian territories.

**Table 2:** Profile of the survey participants.

Total number of respondents	N = 572
**Gender**	
Male	244
Female	328
**Education**	
Undergraduate/Graduate/ Postgraduate and above/ Others	24/208/327/13
**Area of residence**	
Rural/ Urban/Town	108/386/78
**History of COVID infection**	
Infected/Non- infected	198/374
**Regional distribution of responses**	
North	50
West	100
South	274
East	77
North east	18
Central	53

#### Perception of the participants about the various immunity boosting food items to prevent COVID-19

**Fig 3:**
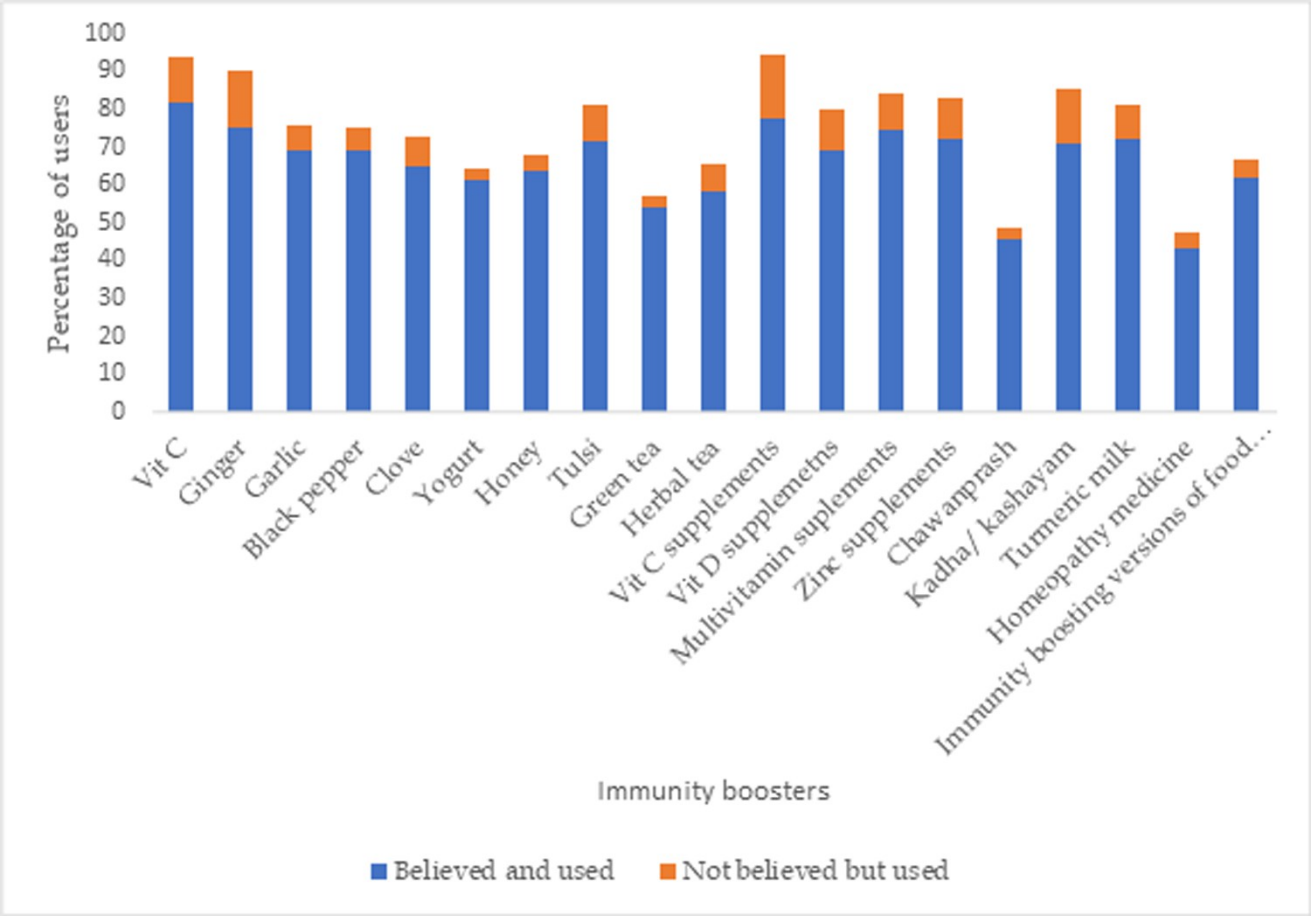
The perception of the respondents who reported to use each of these ‘immunity boosters’ about their ability to prevent COVID-19 infection.

The concept of “immunity boosting foods” as a preventive strategy to fight COVID-19 infection gained a lot of traction during the pandemic. Out of the commonly searched immunity boosting agents, most respondents (71.9%) reported to have increased their consumption of Vitamin C rich foods (citrus fruits, guava, amla etc.) as immunity boosters during the study period. A large proportion of respondents also reported consumption of nutraceuticals supplements such as Vitamin C supplements (68.2%), Zinc supplements (61.4%) to boost immunity. Traditional Indian spices like ginger and garlic were used by 62.9% and 50.9% respondents respectively. Although ‘kadha/kashayam’ (decoction of medicinal herbs) and ’chawanprash’ (an Ayurvedic health mixture made of various herbs and spices) were quite hyped by fewer participants reported to have consumed them (28.8%and 57.5% respectively). Dependence on homeopathy medicines for immunity boosting against COVID-19 was found to be the least (28.1%). The perception of the respondents who reported to use each of these ‘immunity boosters’ about their ability to prevent COVID-19 infection is given in **Fig 3**.

#### Food scares, concerns and food safety practices induced by COVID-19 infodemic

In the pre-COVID times, most respondents reported to have used “lukewarm water “or “water” to clean vegetables and fruits. However, the safety measures taken to avoid spread of COVID-19 through food items changed during the COVID period. Use of special cleaners to wash vegetables and fruits to “remove dust and germs” was reported to be relatively higher during the COVID times than the pre-COVID times. However, some respondents noted a change in the quality, colour and shelf-life of fruits and vegetables after using vegetable cleaners.

**Fig 4:**
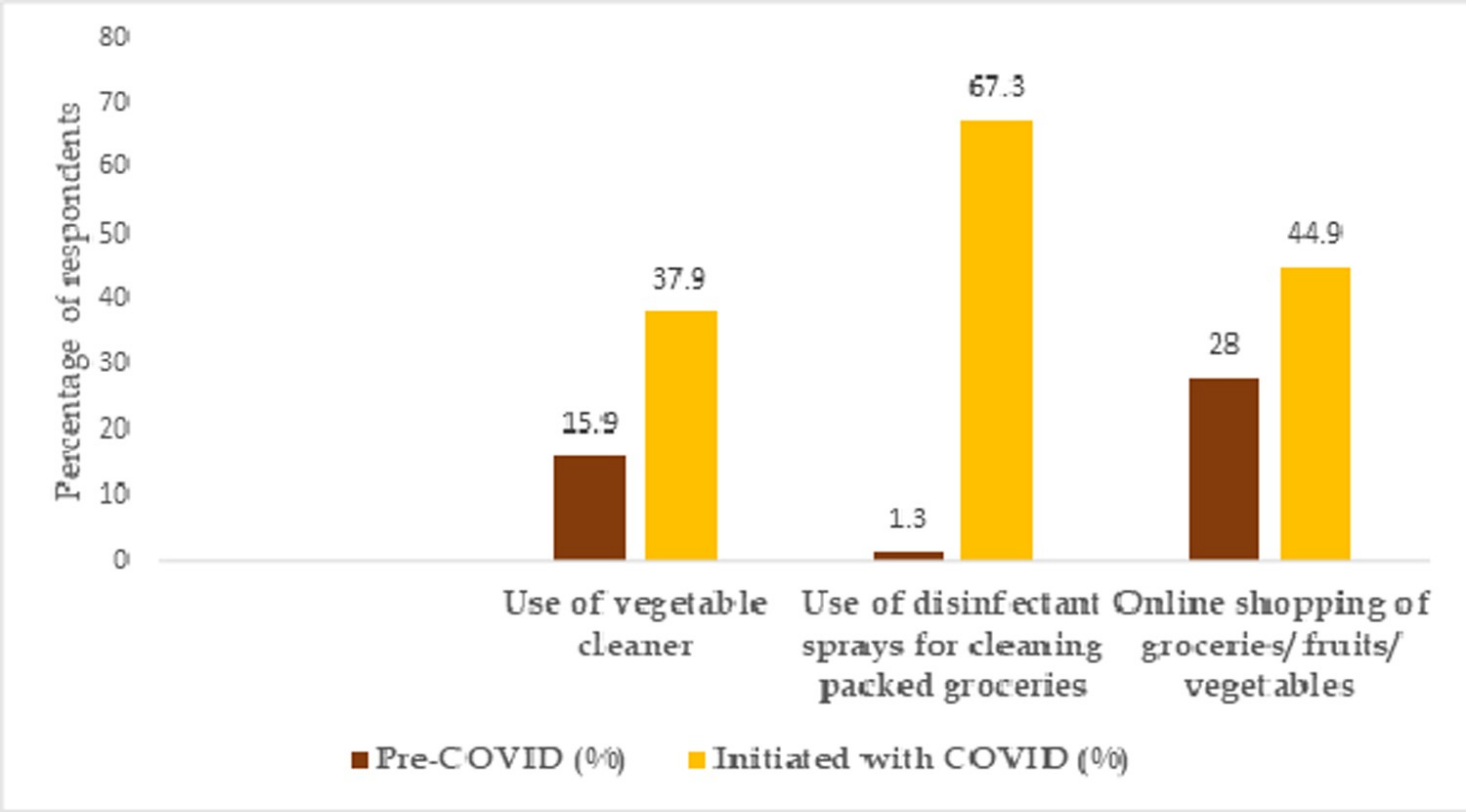
The food safety practices picked up or escalated during the COVID period.

While approximately 60% of the respondents reported to have reduced the frequency of ordering cooked foods via online platforms, the preference to buy vegetables, fruits and groceries from online delivery services increased significantly (p = 0.000) during this time. Out of the 264 non-vegetarian participants, 131 reported that they changed their usual dietary preferences. Overall, 175 participants reported to have had concerns about consuming or ordering Chinese cuisine. However, a majority (83%) showed no concerns about safety of foods in restaurants or eateries in the post lockdown period. The food safety practices picked up during the COVID period probably due to the abundance of information regarding the same is given in **Fig 4.**

### Reliability of different sources of information

**Fig 5:**
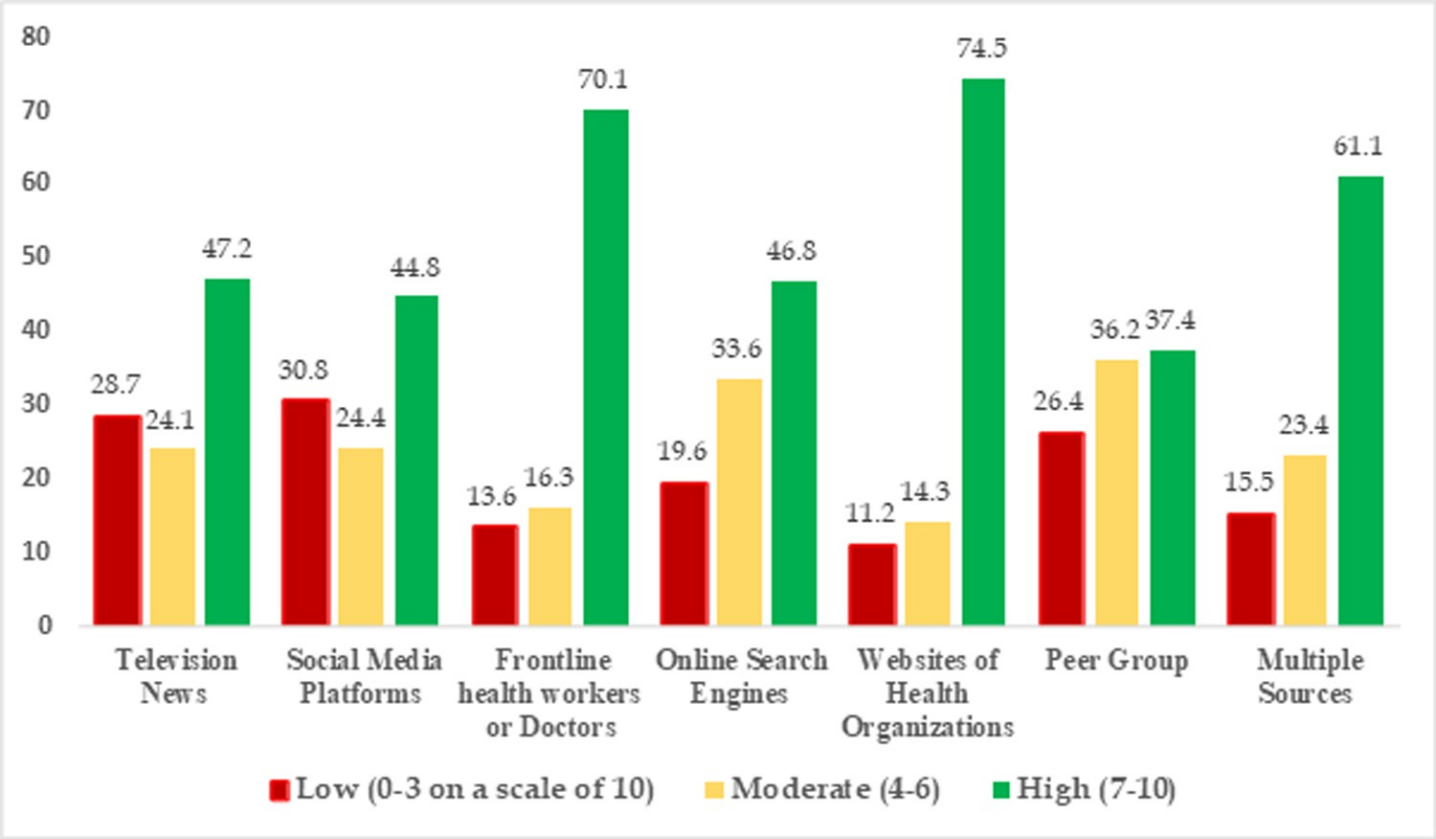
Perceived reliability (%) of various sources among the participants (n = 572) for seeking information on food and nutrition during COVID-19.

The participants sourced most information related to food behavior such as boosting immunity through natural food sources, alternative medicines and home remedies from social media. However, in terms of reliability they ranked health workers and websites of health organizations high (**Fig 5**). Out of the 61.8% respondents who had a history of COVID- 19 infection and were under home isolation, many reported that they relied on the dietary advice provided by doctors and health professionals more than the general information provided by peers, media, and web during their active infection phase. For information related to supplementation, respondents relied on the frontline workers, whereas, for information related to immunity building products social media was the most sought-after source of information.

## Discussion

The pandemic hit the world so suddenly that there were no defined treatment protocols and as a result, people approached various platforms for information about prevention and treatment of COVID-19 disease. This study investigated the food and nutrition related web search behavior of adult Indian internet users during the different phases of the COVID-19 pandemic in India. This study clearly demonstrates that there was a heightened internet search for terms like “immunity”, “supplements”, “vegetable cleaners” etc. during the period. The rise in the COVID-19 cases had a direct association with the search trends. Despite the hype on different media platforms about immunity boosting foods to keep the infection at bay, there has been limited scientific evidence about the efficacy of immunity boosters and an exaggerated immune response as a preventive measure against COVID-19 [16]. The RSV of search terms “Limcee”, ‘Zincovit” which are Indian brands of Vitamin C and Zinc supplements, were found to be high and their search trends coincide with the unprecedented demand for these products in the corresponding time frame in India [17]. The Indian media reported that the Indian market was as much flooded with information as the commercial immunity boosting products such as immune boosting teas, oils, herbal supplements [17]. In this study, a large proportion of the respondents perceived that various supplements and common Indian herbs would help boost their immunity and reported high usage of those. In a study about the effect COVID-19 on consumers’ purchasing patterns, Prasad et al (2021) stated that the fear of contracting COVID-19 led to the consumers to prioritize healthy foods and vitamin supplements [18]. News reports of the corresponding periods in India also reported about the nutraceutical supplements and other immunity boosting products ‘flying off store shelves’ [17]. This credence may have been natural since there was no specific treatment or prevention of COVID-19 at that time.

Though the search interest in Ayurvedic Kadha (a decoction of spices and herbs) was found to be very high, the usage was markedly low. The search trend of the keyword “Ayush Kadha” on Google was found to appear for the first time during the month April 2020. This is probably because of interest evoked by the advocacy about the same by the Indian Prime minister in his national address during a TV appearance on 14^th^ April 2020, wherein he shared tips to build immunity against COVID-19 as recommended by the Ministry of AYUSH (Ayurveda, Yoga & Naturopathy, Unani, Siddha and Homeopathy). However, it’s low usage may be because of its bitter taste that would have made people cringe away from its consumption [19].

In various Indian media, news of spread of the COVID-19 virus through packed foods, scares related to food deliveries, groceries were circulated, which seemed to have impacted people’s perception. Particularly, the news related to a pizza delivery boy in Delhi testing positive for COVID-19 and putting 72 families to whom he delivered pizza at the risk of contracting the disease created a huge buzz throughout the country [20]. The same has been reflected in the Google search trends in the corresponding time frame, where the RSV for the terms like “food delivery”, “zomato”, “street food”, “junk food” were found to be negatively correlated with the growing number of COVID-19 cases. While google search regarding ‘grocery delivery’ spiked during the months of March-August 2020—during the first wave of COVID 19 in India and again during January-March 2021 coinciding with the upsurge of the second wave.

The study participants also reported reduction in frequency of ordering cooked foods through online delivery platforms during the COVID-19 period in comparison to the pre-COVID times. There were concerns about the virus spreading through meat eating, and consumption of poultry, eggs and seafood as well as imported frozen foods, eating cold substances like ice creams [1]. Exposure to such news items seems to have led to the significant change in dietary preferences of most participants from non-vegetarian to vegetarian foods as noted in the study. Public dubiosity about the safety of consumption of non-vegetarian foods, frozen foods and Chinese cuisine is also evident from the common queries made on google like ‘Can I get Coronavirus from frozen foods?’ or increasing search trends on ‘Coronavirus in chicken’. The reported aversions developed against Chinese foods might be because of the public outrage triggered against China among Indian social media users while fighting the ‘China virus’ [21].

The risk of getting COVID-19 from packaging is not clear (FAO and WHO, 2020), yet concerns were raised regarding the transmission of the virus through fomites on food items and food packages. These concerns led to many unscientific practices like keeping the packed foods including the perishable ones isolated for more than 24-hrs, which possibly could have increased the risk of contamination with other food borne microorganisms [1]. In this study, the respondents reported the use of special cleaners to disinfect groceries, fruits and vegetables and also mentioned about the changes observed in the food quality upon using them (such as “change in colour” after using the special cleaners, “difficulty in storing the vegetables” and “shortened shelf life”). However, according to EPA (Environmental Protection Agency, 2020) the instructions on the labels of the disinfectants must be checked for the suggested contact time, suitable surface for application and concentration before they are used. Whereas, global public health authorities have mainly emphasized on the use of PPE (Personal Protective Equipment), hand-washing and physical distancing during food handling rather than use of any special cleaners (FAO and WHO, 2020; FDA, food safety and coronavirus disease, 2020).

Stress related to isolation, quarantine or risk of infection provoked people to gather more information about the disease to which the internet and social media provided an easy and unprecedented access to a great amount of (mis)information. In this study, respondents reported that they largely relied on social media for COVID-19 related information. The (mis)information definitely impacted the social perceptions and augmented the already challenging COVID-19 situation in the country by sometimes distorting accurate scientific guidance, exploiting emotions and creating taboos, scares and biases among common people. Government took efforts to fight the infodemic and India joined the ‘‘#VERIFIED” campaign of the United Nations to counter misinformation and ensure the spread of fact-based content related to the COVID-19 pandemic [22, 23]. However, the responsibility of fighting the infodemic not only pivots on the health organizations and governments but also on the information consumers. As suggested by Johnson (2012), instead of becoming ‘obese’ with the abundance of information, common people should go on ‘information diet’ and be selective about the information that they consume just as we should be selective about the food we eat [2].

### Strengths and limitations

The online survey methodology used to conduct this study may be regarded both as is both the strength and a limitation of the study. During the epidemic situation when conducting in-person surveys was unsafe and challenging, web-based survey was a viable mode of research to gather quick response in real time. With wide usage, dependence and high rates of mobile literacy among all age groups in India, obtaining response from a diverse population from all across the country had been possible through this mode of survey. Although, the lesser number of respondents taking the survey may limit the generalizability of the study findings. This low response rate of web surveys has been highlighted as a major limitation of by various studies [24, 25]. However, while designing the study tool certain factors possibly impacting(decreasing) study response rate or quality of survey response such as time-consuming survey questionnaire, survey questionnaire being marked as junk/spam when sent through email, incentives, sample recruitment strategy, solicitation mode [26] were taken care of. The study questionnaire pertained to the average attention span of respondents and instead of being invited through emails, voluntary response through open access government website were seeked, no incentives were offered. Hence it is expected that the low response rate in this study might have not led to response bias.

The findings of our study highlight the need for promoting media literacy and health literacy among the people so as to advocate for use of information cautiously, verifying the authenticity and accuracy of health information before putting it to practice or sharing it with others. Nutrition literacy, media literacy as a part of national programs can help in developing skills among common people to rationalize circulating information without worry of another future infodemic. These should be a part of the country’s preparedness for facing the challenge of future ‘infodemic in pandemic’.
